# An Investigation into the Public’s Attitude Toward Opting out of Brain Death

**DOI:** 10.1007/s12028-024-02196-8

**Published:** 2025-01-14

**Authors:** Nicholas Ludka, Deidre Hurse, Abram Brummett

**Affiliations:** 1 Department of Foundational Medical Studies, Oakland University William Beaumont School of Medicine, Rochester, MI USA; 2 Clinical Ethics, William Beaumont University Hospital, Royal Oak, MI USA

**Keywords:** Brain death, Death, Decision-making (shared), Bioethics

## Abstract

**Background:**

There have been growing sentiments that the Uniform Determination of Death Act needs to be revised. One suggestion is to include a conscience clause, that is, allowing patients to “opt-out” of brain death determination. Understanding public attitudes toward a conscience clause may help inform policymakers and future proposed revisions. Therefore, we sought to investigate informed public attitudes toward continued medical support after the determination of brain death.

**Methods:**

A nationwide online survey was distributed by a third-party provider. The survey had three components: (1) a 2-min educational video that explains five basic facts of brain death, (2) a validated five-item questionnaire to measure understanding of brain death, and (3) a six-item questionnaire to measure informed public attitudes toward a family’s request to continue medical support for a patient with brain death. Attitudes were measured on a seven-point Likert scale. A multiple linear regression model was developed to identify predictors of attitudes toward opting out of brain death. Analysis of variance with a post hoc Tukey test was used to compare attitudes across categorical demographic variables.

**Results:**

We collected 1386 responses from participants across 49 states. The average five-item knowledge score was 88%. A total of 41.9% of all participants agreed that the hospital should be required to continue treatment for an individual with brain death if their family rejects brain death. A total of 24.4% and 27.3% of participants would request further treatment for themselves and a family member after a determination of brain death, respectively. Multiple linear regression identified attitudes for oneself and for a family member, age greater than 65 years, understanding that brain death is legal death, and male sex as predictors of attitudes toward requiring continued treatment (F(6, 1380) = 142.74, adjust *R*^2^ = 0.38, *p* < 0.001).

**Conclusions:**

Nearly half of the participants would require hospitals to continue treatment for families who reject brain death as death. Future discussions on revising the Uniform Determination of Death Act to adopt a conscience clause should consider informed public attitudes.

**Supplementary Information:**

The online version contains supplementary material available at 10.1007/s12028-024-02196-8.

## Introduction

Brain death was formalized as the medico-legal standard of death in 1981 when the President’s Commission for the Study of Ethical Problems in Medicine and Biomedical and Behavioral Research created the Uniform Determination of Death Act (UDDA). The UDDA states that death is declared when an individual has sustained either (1) irreversible cessation of circulatory and respiratory functions or (2) irreversible cessation of all functions of the brain, including the brain stem [1]. The UDDA was created in response to the growing patchwork of state statutes across the United States that were created in the wake of the *Definition of Irreversible Coma* report by the Ad Hoc Committee of the Harvard Medical School to Examine Brain Death [2]. The President’s Commission sought to create a uniform recommendation to remedy the growing disparities between states as well as give justification for the whole-brain and cardiopulmonary views of death. Although some deviation exists across states, the UDDA has been widely adopted as the medico-legal standard across all 50 states and is endorsed by various medical and legal organizations [3].

The most notable deviation from the UDDA is in New Jersey, where an individual can “opt-out” of brain death determination and instead require physicians to use the cardiopulmonary definition if the individual has a religious-based disagreement with brain death [4]. In these cases, continued support is provided after brain death determination until there is death by cardiopulmonary criteria (e.g., after irreversible cardiac arrest). Patients with brain death who continue to receive treatment could potentially have a heartbeat for months after a determination of brain death [5]. The New Jersey exemption stands alone as the only statute in the United States that requires hospitals to continue indefinite support. However, New York [6] and California [7], while not requiring indefinite support, have “reasonable accommodation” clauses requiring a period of continued support for families with an understanding that support will be withdrawn after this period has ended. Illinois adds leniency to their death statute by requiring hospitals to adopt policies and procedures to allow health care professionals, in documenting a patient’s time of death at the hospital, to take into account the patient’s religious beliefs concerning the patient’s time of death [8]. This allows families time to accept that their loved one is dead, even when their cardiopulmonary function continues.

Public understanding of the basic facts of brain death remains poor in the United States. For example, the survey by Siminoff et al. [9] demonstrated that 28.1% of the public believes that people with brain death can still listen to their surroundings. Limited familiarity with the basic facts of brain death may be due to inaccurate media portrayals of brain death, in which politicians are described as “brain dead” or patients are declared brain dead but kept alive on *life* support [10]. Contradictory or misleading statements confuse the public and their perception of a foundational medico-legal concept. Such variations in understanding may lead to objections to brain death, not because they have a religious objection, but because they misunderstand the patient to be alive [11].

Although the UDDA has been in place for 40 years, there has been a growing sentiment that it needs to be changed [12–16]. In response to these concerns, the Uniform Law Commission (ULC) created a drafting committee in 2021 to discuss changes to the UDDA [17]. One proposal raised during the discussions was to adopt an expanded New Jersey–style “conscience clause” that would allow patients and families to opt-out of brain death for both religious and philosophical reasons. The ULC received comments from medical, organ procurement, and advocacy organizations throughout 2023 on multiple proposed changes to the UDDA [18]. Religious organizations such as Life Guardian Foundation and the Catholic Medical Association advocated for a conscience clause on the basis of freedom of religion. In contrast, secular organizations such as the Terry Schiavo Life and Hope Network and the Pacific Justice Institute defended a conscience clause based on medical (i.e., potential for inaccurate brain death diagnosis) and legal (i.e., due process for surrogate) reasons, respectively. On the other hand, many organizations, from the American Society of Transplant Surgeons to state hospital associations (Alabama, Arizona, Texas, and Wisconsin) advocated against including a conscience clause in a revised UDDA. Ultimately, a consensus position could not be reached, causing the ULC to pause discussions in the fall of 2023.

The ULC leadership published an article in early 2024 describing the deliberations, saying that, “We believe that a pause to the existing efforts to revise the UDDA allows democratic deliberation to continue over whether broadly accepted statutory revisions to determinations of death can be achieved” [19]. A key piece of this democratic process is considering the beliefs and attitudes of the general public. Although a substantial proportion of the public now recognizes the medical definition of brain death, there remains a significant divergence in how it is accepted across different cultural and moral beliefs, but the extent of this divergence is not well studied. Furthermore, although public attitudes toward brain death have been published elsewhere, they may be influenced by varying degrees of knowledge about brain death, that is, the data are not an accurate description of *informed* attitudes toward brain death because the respondents were not *educated* about the nuances of brain death before soliciting their attitudes [20]. Much of the literature is focused on brain death attitudes as it is applied to organ transplantation, which may confound an individual’s attitudes toward a particular view of death [9, 21–25]. Therefore, we sought to study informed public attitudes toward brain death and a conscience clause, that is, attitudes following an educational intervention, to clarify common misunderstandings (e.g., that brain death is reversible). Although the current effort to revise the UDDA has been paused, future efforts at revision that will consider adding a conscience clause for brain death may be forthcoming. This study seeks to inform policymakers on the public attitudes toward and use of a conscience clause for brain death.

## Methods

### Survey Development

The survey included an educational video (access here), a five-item questionnaire to test understanding of brain death, and a six-item attitudes questionnaire. The video provided a detailed overview of five fundamental concepts related to brain death, closely aligning with those outlined in a validated tool used to assess the understanding of brain death among the general public [26]. This tool was developed to cover three core constructs: (1) that patients with brain death are completely apneic and require mechanical ventilation, (2) that brain death is irreversible, and (3) that brain death is distinctive from a persistent vegetative state. Through an iterative process involving focus groups of laypersons and experts in intensive care medicine, neurosurgery, trauma surgery, and psychiatry, Tawil et al. [26] developed a five-item tool that showed test–retest validity (Spearman ρ = 0.72), internal consistency (Cronbach’s α = 0.62), and discriminatory ability between laypersons and experts. After watching the video, the five-item validated tool was delivered. The participants then read the following scenario:

C.H. is a patient in the intensive care unit at the local hospital. Three days ago, C.H. suffered a significant brain injury. A breathing machine is keeping C.H.’s lungs working, and medications are being used to maintain C.H.’s other organs. A neurologist is consulted to determine the extent of C.H.’s brain injury. The neurologist’s examination shows that C.H. is unconscious, cannot breathe without the ventilator, and doesn’t have any brainstem reflexes. Based on these results, the neurologist determines that C.H. is brain dead, and, further, that C.H. will never recover any of these functions. When the neurologist meets with C.H.’s family to tell them that C.H. is brain dead, the family objects and says that C.H. isn’t dead until the heart stops beating.

The scenario was intentionally written to be age-neutral and gender-neutral, and the mechanism of injury was not specified. Framing biases may influence an individual’s attitudes, and avoiding such language helps focus the reader’s attention on the core subject (i.e., brain death) and away from details that, although important (e.g., age of patient), may distract from the core subject. It also includes key facts (e.g., apnea and areflexia) demonstrated in the educational video and tested using the validated tool. Participants were first asked whether the patient in the vignette was dead according to current U.S. laws. They then completed a six-item questionnaire to assess their attitudes across three subjects: continuing treatment after a determination of brain death for oneself, a family member, and a stranger; who ought to pay for continued treatment; and what types of interventions ought to be offered to an individual after a determination of brain death. Attitude items were chosen based on previous surveys, suggested gaps in the literature, and issues identified in the medical and bioethics literature that attend to brain death. Attitudes were measured on a seven-point Likert scale (1 = strongly disagree to 7 = strongly agree).

Participants were also asked to provide the following demographic information: age, sex, zip code, income bracket, highest education level, political affiliation, race and ethnicity, religious affiliation, and religious commitment. Zip code data were then used to group participants into geographic regions outlined in the U.S census. The regions were as follows: Northeast: CT, ME, MA, NH, RI, VT, NJ, NY, and PA; Midwest: IN, Ill, IA, KS, MI, MN, MO, NE, ND, OH, SD, and WI; South: AL, AR, DE, District of Columbia, FL, KY, GA, LA, MD, MS, NC, OK, SC, TN, TX, VA, and WV; and West: AK, AZ, CA, CO, HI, ID, NM, NV, OR, UT, WA, and WY. Religious commitment was measured on a four-point Likert scale (1 = not committed to 4 = very committed). Before distribution, the survey was sent to 10 experts on clinical ethics and brain death. Feedback on the clinical vignette and attitude measures was collected and implemented before final distribution. The survey and participant information sheet can be found in the Supplemental Materials. All participants provided informed consent electronically before participating in the survey. This study was approved by the Oakland University Institutional Review Board IRB-FY2023-34.

### Survey Distribution

The researchers used Oakland University’s Qualtrics platform to design an online survey distributed via Centiment. Participants voluntarily create profiles on Centiment through social media ads, search engines, or website partnerships. The research team did not directly recruit; instead, Centiment recruited respondents and provided all incentives through their platform. Before signing up, respondents are informed that incentives are dynamic. For this study, respondents were primarily compensated directly, with some opting to donate their incentives to a local school or nonprofit of their choice. The monetary value of incentives for respondents ranged from approximately $1 to $2.57, depending on the dynamic reward system used by the platform. Before the study was sent to respondents, their profiles were screened by Centiment to ensure they met specific demographic and psychographic inclusion requirements for the study. These requirements included being in the United States, being 18 years or older, and being established as part of Centiment’s panel of respondents. The target number of participants was established before dissemination and based on previous survey studies on public attitudes and knowledge of brain death [9, 22, 27, 28]. Before participating in the survey, respondents were only informed about the estimated 12-min duration of the survey. This was done to prevent selection bias until respondents showed interest in the study. Data collection began in October 2023 and concluded in November 2023. The process was temporarily paused at around 50% completion to perform an interim analysis of respondents’ racial and ethnic demographics. This analysis, conducted with a Centiment representative, confirmed that the sample aligned with recent census data, ensuring demographic representativeness. Because the racial and ethnic distribution met expectations, no changes to the survey distribution method were necessary. Data collection then resumed, and the target number of participants was achieved within the planned time frame. We employed listwise deletion to handle missing data, ensuring that only cases with complete responses across all variables were included in the final analysis. Centiment uses IP addresses tracking, unique survey links, and algorithms to detect duplicate responses suggestive of manipulation, and an invisible ReCaptcha to block bots and fraudulent attempts.

### Statistical Analysis

Answers to the five-item questionnaire are reported as a percentage of correct responses. Descriptive statistics are reported as frequency and percent. Mean scores ($\overline{x }$), difference in means (${\overline{x} }_{diff}$), and standard deviations (SDs) are reported where appropriate. Analysis of variance (ANOVA) tests with Tukey’s post hoc analysis was used to compare mean scores across groups, and analysis of covariance was performed, when appropriate, to control for covariates. Bonferroni corrections were performed for multiple pairwise comparisons. The assumption of equality of variances was confirmed with Levene’s test. *χ*^2^ tests were used to compare categorical variables. Multivariable linear regression on the outcome variable (Likert scale score of whether the hospital is required to continue treatment after a determination of brain death for the patient in the vignette) was performed as previously described [28]. It included the following predictors: age, race and ethnicity, religion, sex, religious commitment, income level, highest level of education, five-item quiz score, identifying the patient in the vignette as legally dead, political affiliation, requesting further treatment for oneself, and requesting further treatment for a family member. Backward selection of independent variables with a threshold of *p* < 0.05 to remain in the model yielded six predictor variables. The tolerance threshold was set at 0.10 to test for collinearity, and regression of residuals was confirmed with the Durbin-Watson statistic. The results from the reduced model are presented here. Analysis was performed using SPSS 29.0.1 software. All hypotheses were two-sided, and the α was set a priori at 0.05.

## Results

### Participant Demographics

**Fig. 1:**
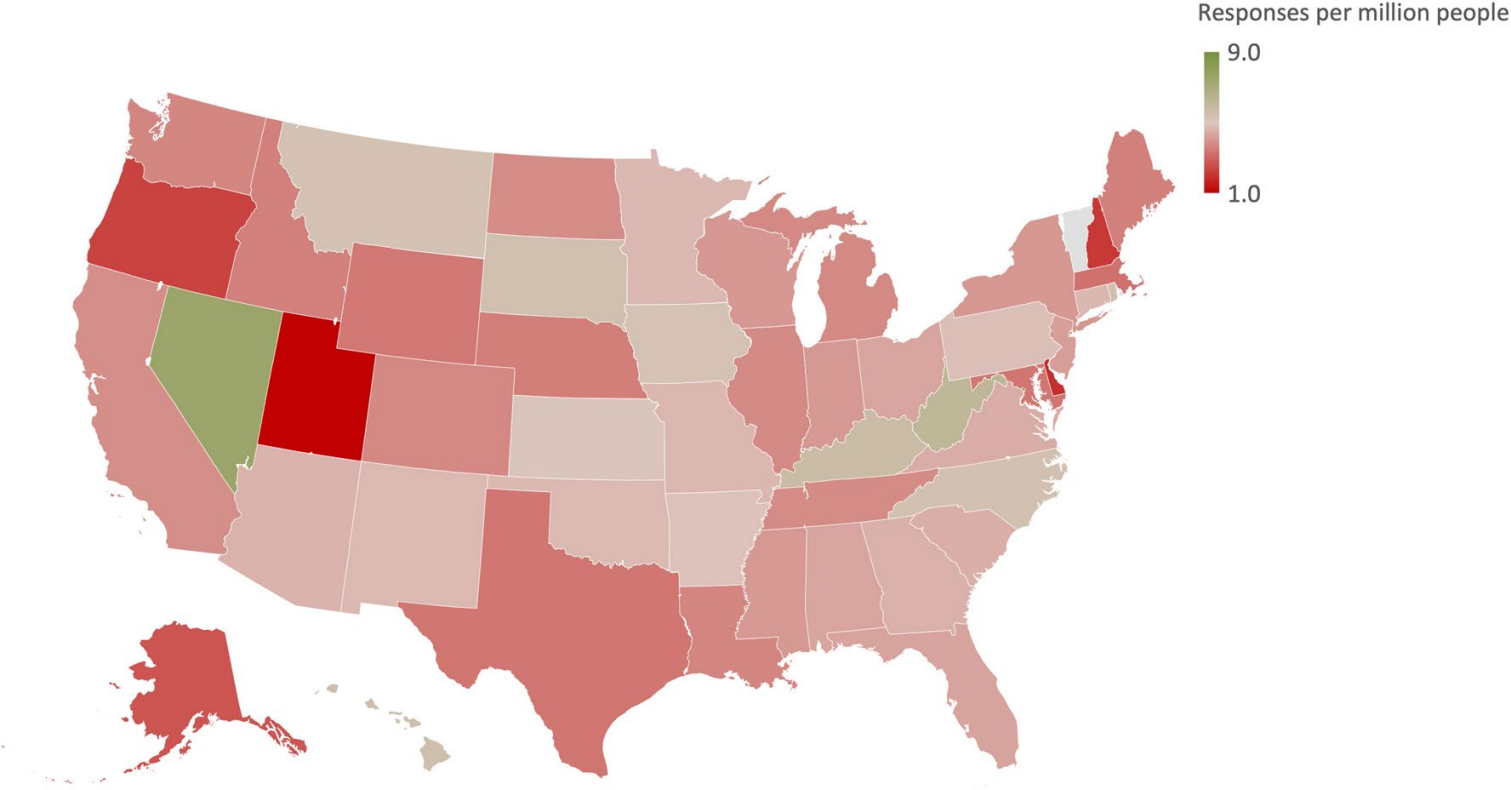
Participant distribution across United States normalized to state population [29]

Of 1768 surveys with at least one response, 1386 (78%) were completed and included in the final analysis. Participants lived in all regions of the United States except the state of Vermont (Fig. 1). The greatest proportion (39.4%) of respondents were located in the South. The average age was 49.0 years (SD = 17.2 years). The population was approximately equal across men and women. The majority (57.1%) of respondents had an income greater than $50,000 (Table 1). The highest level of education achieved by most participants was less than college (36.9%), whereas 38.6% of participants had at least an undergraduate degree. There were approximately equal numbers of Democrats (34.6%) and Republicans (33.2%), with smaller proportions of Independents (24.8%) and those with no political affiliation (6.7%). The majority of respondents (73.8%) were White. A variety of religions were represented, with the most common being Catholicism (20.5%), Protestantism (18.2%), and other Christian (20.6%), with one quarter of respondents reporting no affiliation. More than two thirds (66.9%) of those with a religious affiliation reported being at least moderately committed to their religion.

**Table 1:** Demographic information of participants (n = 1386)

	N (%)
Region	
Northeast	236 (17.0)
Midwest	293 (21.2)
South	547 (39.4)
West	311 (22.4)
Gender	
Male	710 (51.2)
Female	671 (48.1)
Transgender	5 (0.4)
Age	
18–24	108 (7.8)
25–34	329 (17.2)
35–44	265 (19.1)
45–54	227 (16.4)
55–64	248 (17.9)
> 65	300 (21.6)
Religious Affiliation	
Christian	
Catholicism	284 (20.5)
Church of Jesus Christ of Latter-Day Saints	253 (18.2)
Protestantism	44 (3.2)
Other Christian	286 (20.6)
Judaism	18 (1.3)
Islam	36 (2.6)
Buddhism	36 (2.6)
Other	78 (5.6)
No affiliation	351 (25.3)
Religious Commitment	
Not Committed	512 (36.9)
Slightly Committed	340 (24.5)
Moderately Committed	317 (22.9)
Very Committed	218 (15.7)
Political Affiliation	
Republican	460 (33.2)
Democrat	480 (34.6)
Independent	344 (24.8)
No affiliation	93 (6.7)
Other	9 (0.6)
Race/Ethnicity	
Caucasian or White	1023 (73.8)
African American or Black	215 (15.5)
Latina or Hispanic	109 (7.9)
Asian	61 (4.4)
Indigenous or Native American	19 (1.4)
Middle Eastern or Northern African Descent	5 (0.4)
Native Hawaiian or Pacific Islander	5 (0.4)
Income Level ($)	
< 24,999	268 (19.1)
25,000 – 49,999	325 (23.4)
50,000 – 99,999	478 (34.5)
100,000 +	314 (22.6)
Highest Level of Education	
Less than college	512 (36.9)
Trade school or associate’s degree	340 (24.5)
Bachelor’s degree	317 (22.9)
Advanced degree	218 (15.7)

## Differences in Understanding of Brain Death

The average number of correct responses across all participants was 4.44 of 5 (SD = 0.92), with 85% of participants answering at least 4 of 5 correctly (Table 2). The question, “Will someone who is brain dead react (grimace, move away, or blink) if someone touches their eyeball?” had the lowest percentage (86.8%) of correct responses. A total of 81.1% of participants correctly identified the patient with brain death depicted in the vignette as dead according to current U.S. laws. Individuals who correctly identified the patient in the vignette as dead had a higher five-item score ($\overline{x }$= 4.56 of 5, SD = 0.81) compared with those who incorrectly identified the patient as alive ($\overline{x }$ = 3.92 of 5, SD = 1.13) (*p* < 0.001).

**Table 2:** Number of responses to 5-item questionnaire after watching educational video

	Yes (%)	No (%)
Can someone who is brain dead breathe without the support of a breathing machine?	149 (10.7)	1237 (89.3)
Can someone who is brain dead ever wake up (recover)?	173 (12.5)	1213 (87.5)
Will someone who is brain dead react (grimace, move away, or blink) if someone touches their eyeball?	182 (13.2)	1204 (86.8)
Can a person be brain dead even if their heart is beating?	1228 (88.6)	158 (11.4)
Is brain death different from a coma or vegetative state?	1273 (91.8)	113 (8.2)

### Region

Respondents from the Midwest had significantly higher quiz scores (${\overline{x} }_{diff}$ = 0.24, 95% confidence interval [CI] 0.03–0.44) compared with respondents from the Northeast despite having comparable correct responses to identifying the patient as dead according to U.S. laws (*χ*^2^ = 1.94; df = 1, *p* = 0.16) (Table 3). There were no differences between the South, Midwest, and West.

**Table 3:** Descriptive statistics across demographic variables for quiz score and percentage of participants who correctly identified the brain-dead patient as dead according to U.S. laws

	Average quiz score (SD)	Correct identification (%)
Region	*	N.S
Northeast	4.34 (1.02)	76.3
Midwest	4.58 (0.76)	81.2
South	4.43 (0.94)	83.0
West	4.40 (0.93)	81.4
Gender	*	N.S
Male	4.38 (0.98)	81.3
Female	4.50 (0.85)	81.2
Transgender	5.00 (0.00)	40.0
Age	***	***
18–24	4.11 (1.16)	71.3
25–34	4.26 (1.04)	67.4
35–44	4.25 (1.05)	78.1
45–54	4.40 (0.91)	81.9
55–64	4.66 (0.66)	88.7
65 +	4.72 (0.62)	91.0
Religious Affiliation	***	N.S
@Christian		
Catholicism	4.33 (1.00)	78.9
Church of Jesus Christ Latter-day Saints	3.86 (1.25)	77.8
Protestantism	4.64 (0.74)	85.4
Other Christian	4.44 (0.95)	82.5
@Judaism	4.55 (0.82)	84.1
@Islam	3.47 (1.21)	66.7
@Buddhism	4.39 (0.98)	88.9
No Affiliation	4.51 (0.81)	81.5
Political Affiliation	**	N.S
Democrat	4.31 (1.03)	78.3
Republican	4.50 (0.85)	82.4
Independent	4.51 (0.85)	82.0
No Affiliation	4.53 (0.87)	86.0
Race/Ethnicity	***	N.S
Caucasian or White	4.52 (0.86)	84.0
African American or Black	4.16 (1.07)	69.0
Latina or Hispanic	4.07 (1.03)	79.8
Asian	4.54 (0.95)	63.9
Indigenous or Native American	4.54 (0.95)	85.4
Native Hawaiian or Pacific Islander	4.57 (1.13)	100
Middle Eastern or North African Descent	4.43 (0.79)	71.4
Income Level ($)	N.S	N.S
< 24,999	4.37 (0.93)	79.9
25,000–49,999	4.50 (0.87)	81.2
50,000–99,999	4.48 (0.87)	83.9
> 100,000	4.39 (1.02)	78.0
Highest Level of Education	***	N.S
Less than college	4.41 (0.92)	79.2
Trade school or associate’s degree	4.56 (0.81)	83.5
Bachelor’s degree	4.49 (0.86)	79.8
Advanced degree	4.24 (1.11)	82.6

Quiz scores reported as average score (SD)

1 – Strongly disagree, 2 – Disagree, 3 – Somewhat disagree, 4 – Neither agree nor disagree, 5 – Somewhat agree, 6 – Agree, 7 – Strongly agree

Statistics for quiz score calculated using one-way ANOVA test, *p < .05, **p < .01, ***p < .001

Statistics for correct identification calculated using Chi-square test,

N.S., not significant

### Sex

There were differences in five-item questionnaire scores between sexes (F(2, 1384) = 3.77, *p* = 0.023), and post hoc analysis showed that female scores were slightly higher than male scores (${\overline{x} }_{diff}$ = 0.12, 95% CI 0.01–0.28). However, there were no differences in the proportion of men (81.3%) and women (81.2%) who correctly identified the patient as dead according to U.S. laws (Table 3).

### Age

There were differences in quiz scores (F(5, 1381) = 16.26, *p* < 0.001) and proportion of correct identification of legal death (*χ*^2^ = 66.2; df = 5, *p* < 0.001) between age groups. Respondents who were greater than 65 years old had significantly higher quiz scores compared with those in 18–24, 25–34, 35–44, and 45–54 age groups and had significantly greater proportion of correct identifications of brain death as the legal definition of death compared with all other age groups. A similar pattern was seen for respondents in the 55–64 age group, who had higher quiz scores and a greater proportion of correct responses compared with 18–24, 25–34, 35–44, and 45–54 age groups (Table 3). There were no differences in quiz scores between 18–24, 25–34, 35–44, and 45–54 age groups.

### Religion

There were also differences between religions (F(8, 1378) = 9.78, *p* < 0.001). Islamic respondents had lower quiz scores compared with all religions, with the exception of the Church of Jesus Christ Latter-day Saints, in which quiz scores were comparable (${\overline{x} }_{diff}$ = − 0.39, 95% CI − 1.04 to 0.27) (Table 3). Although there was no group difference in the number of correct responses for identifying a patient with brain death as dead (*χ*^2^ = 13.76; df = 8, *p* = 0.09), post hoc analysis showed that Islamic participants had a lower proportion of correct responses compared with all religions.

### Political Affiliation

There were group differences in quiz scores between political affiliations (F(3, 1383) = 4.53, *p* < 0.01). Post hoc analysis showed that Democratic respondents had lower quiz scores compared with Republicans (${\overline{x} }_{diff}$ = − 0.18, 95% CI − 0.34 to − 0.03) and Independents (${\overline{x} }_{diff}$ = − 0.19, 95% CI − 0.36 to − 0.03).

### Race and Ethnicity

Differences in quiz scores were present between race and ethnicity groups (F(6, 1382) = 6.64, *p* < 0.001), with Asian participants having lower scores compared with White (${\overline{x} }_{diff}$ = − 0.45, 95% CI − 0.80 to − 0.10) and Latina or Hispanic (${\overline{x} }_{diff}$ = − 0.43, 95% CI − 0.86 to − 0.004).

### Income Level

There were no differences in quiz scores (F(3, 1383) = 1.72, *p* = 0.16) or correct identification of brain death between levels of income.

### Education Level

There were significant differences in quiz scores (F(3, 1383) = 6.01, *p* < 0.001) across levels of education despite no differences in the proportion of correct identification of brain death as legal death (*χ*^2^ = 2.81; df = 3, *p* = 0.42). Post hoc analysis showed that respondents whose highest level of education was an advanced degree had lower average quiz scores compared with those whose highest level of education was trade school or an Associate’s degree (${\overline{x} }_{diff}$ = − 0.32, 95% CI − 0.53 to − 0.11) and Bachelor’s degree (${\overline{x} }_{diff}$ = − 0.25, 95% CI − 0.49 to − 0.04).

## Attitudes Toward Continued Treatment for Self, Family, and Stranger

**Fig. 2:**
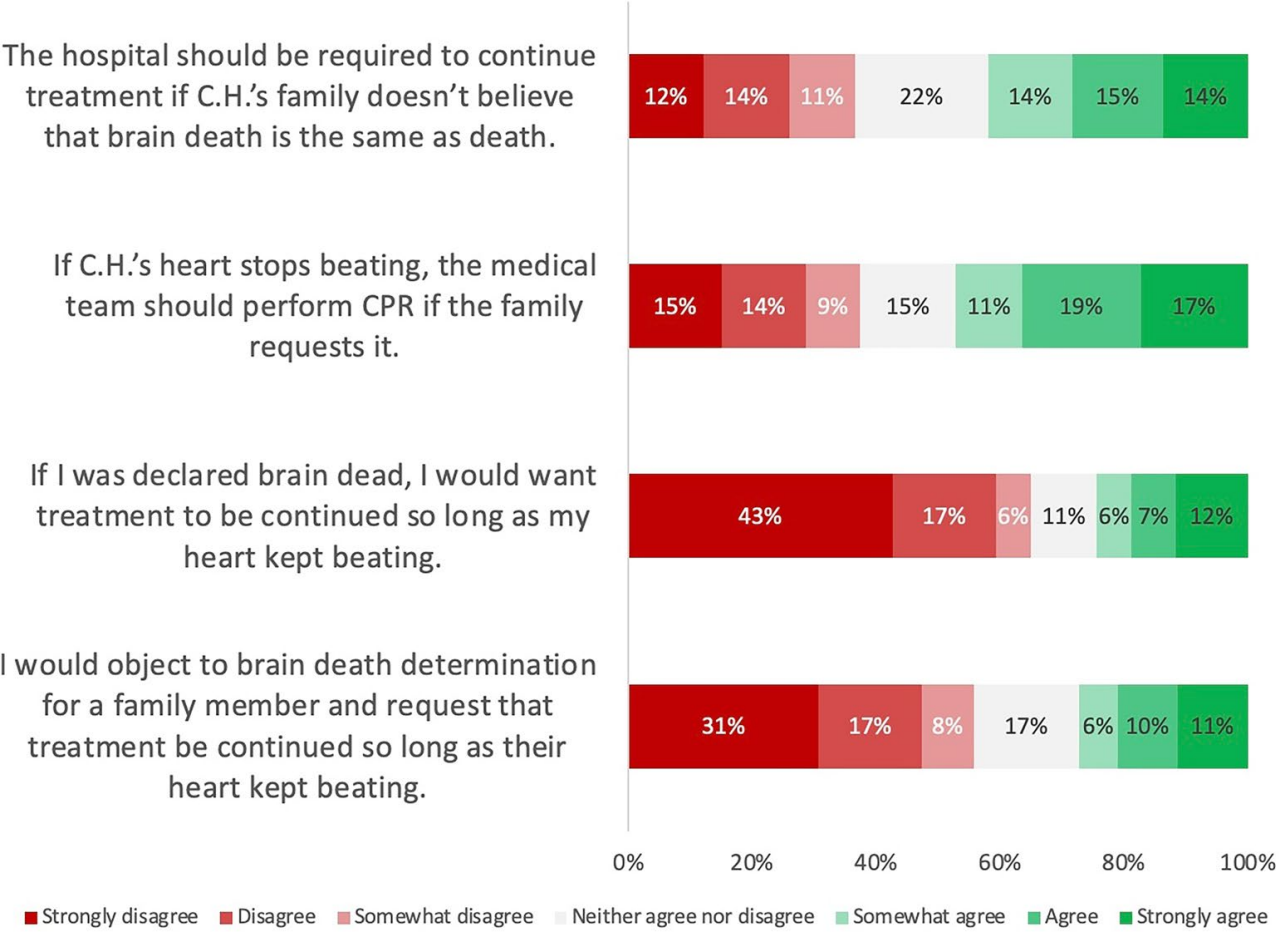
Distribution of responses across a 7-point Likert scale in response to four attitude measures

Nearly half of all participants at least somewhat agreed that the hospital should be required to continue treatment for an individual with brain death if their family does not believe that brain death is equivalent to death (Fig. 2). This contrasts with what participants would choose for themselves or a family member. A total of 24.4% of all participants would at least somewhat agree to further treatment for themselves, with nearly half selecting “strongly disagree” to further treatment (Fig. 2). Similarly, 27.3% of all participants would at least somewhat agree to further treatment for a family member, with more than half selecting “strongly disagree” to further treatment. A similar pattern is seen for cardiopulmonary resuscitation (CPR) for the patient depicted in the vignette, in which 47.2% of all respondents at least somewhat agreed that if the family requests CPR, the medical team should perform it in the event the patient’s heart stopped beating. Even among those who correctly identified the patient as dead according to current medico-legal standards (i.e., brain death), 34.1% at least somewhat agreed that the hospital should be required to continue treatment, and 43.4% at least somewhat agreed that the medical team should perform CPR in the event of cardiac arrest if the family requests it.

## Differences in Attitudes Across Demographic Variables

### Region

There were no regional differences in agreement toward further treatment (F(3, 1383) = 1.39, *p* = 0.25) and CPR (F(3, 1383) = 1.70, *p* = 0.16) for the patient in the vignette, and no differences in agreement for treatment for oneself (F(3, 1383) = 1.89, *p* = 0.13). The only group regional difference came in requesting further treatment for a family member after brain death determination (F(3, 1383) = 3.04, *p* = 0.028). Post hoc analysis showed that respondents from the Midwest had less agreement compared with respondents in the Northeast (${\overline{x} }_{diff}$ = − 0.46, 95% CI − 0.82 to − 0.01) for requesting further treatment for a family member. A state-by-state distribution of responses to the four attitude measures can be found in the Supplemental Information (Fig. S1).

#### Sex

Female respondents had more agreement for further treatment (${\overline{x} }_{diff}$ = 0.23, 95% CI 0.03–0.43) for the patient in the vignette and less agreement for further treatment for a family member (${\overline{x} }_{diff}$ = − 0.24, 95% CI − 0.46 to − 0.02), although the average score among female respondents for further treatment was between “neither agree nor disagree” and “somewhat agree.” There were no differences between men and women for requiring CPR (${\overline{x} }_{diff}$ = − 0.03, 95% CI − 0.25 to 0.20) and requesting further treatment for oneself (${\overline{x} }_{diff}$ = 0.19, 95% CI − 0.04 to 0.42).

#### Age

There were differences between age groups for the requirement to continue treatment for the patient in the vignette (F(5, 1381) = 19.60, *p* < 0.001), to perform CPR on the patient in the vignette (F(5, 1381) = 14.79, *p* < 0.001), to request treatment for oneself (F(5, 1381) = 27.52, *p* < 0.001), and to request treatment for a family member (F(5, 1381) = 28.69, *p* < 0.001). Respondents who were greater than 65 years of age had significantly less agreement on all four measures compared with respondents in the 18–24, 25–34, 35–44, and 45–54 age groups. Similarly, respondents from the 54–65 age bracket had significantly less agreement across all four measures compared with respondents in the 18–24, 25–34, 35–44, and 45–54 age groups. Group differences were still present across all four measures after including quiz scores as a covariate.

#### Religion

Univariate ANOVA indicated a difference between Christian, Buddhist, Jewish, and Islamic participants for the requirement to continue treatment for the patient in the vignette (F(3, 1383) = 5.76, *p* < 0.001), to perform CPR on the patient in the vignette (F(3, 1383) = 4.27, *p* < 0.001), to request treatment for oneself (F(3, 1383) = 13.89, *p* < 0.001), and to request treatment for a family member (F(3, 1383) = 12.69, *p* < 0.001). Analysis of covariance with religious commitment and five-item quiz score as covariates did not change the effect size across all four measures. Islamic respondents had more favorable attitudes (i.e., more agreement for continued treatment after the determination of brain death) compared with Christian and Jewish respondents, corresponding to an average between “somewhat agree” and “agree” on all four attitude measures (Table 4).

**Table 4:** Descriptive statistics for attitudes across demographic variables. Reported as average score (SD)

	Vignette patient			
	Required to provide further treatment	Required to perform CPR	Further treatment for self	Further treatment for family member
Region	N.S	N.S	N.S	*
Northeast	4.31 (1.96)	4.39 (2.08)	3.07 (2.22)	3.41 (2.02)
Midwest	3.99 (1.96)	4.12 (2.06)	2.64 (1.97)	2.94 (2.03)
South	4.05 (1.94)	4.09 (2.13)	2.92 (2.20)	3.35 (2.15)
West	4.11 (1.83)	4.32 (2.06)	2.89 (2.24)	3.28 (2.15)
Gender	*	N.S	*	N.S
Male	3.99 (2.01)	4.18 (2.14)	2.98 (2.25)	3.37 (2.13)
Female	4.21 (1.83)	4.21 (2.04)	2.79 (2.08)	3.14 (2.08)
Transgender	3.00 (1.00)	4.20 (1.93)	1.00 (0.00)	3.00 (2.45)
Age	***	***	***	***
18–24	4.74 (1.74)	4.81 (1.89)	3.79 (2.29)	4.24 (2.13)
25–34	4.47 (1.80)	4.56 (2.00)	3.46 (2.18)	3.82 (2.06)
35–44	4.54 (1.94)	4.69 (2.08)	3.51 (2.36)	3.82 (2.24)
45–54	4.24 (1.84)	4.24 (2.06)	2.91 (2.20)	3.37 (2.15)
55–64	3.75 (1.90)	3.73 (2.09)	2.24 (1.83)	2.57 (1.79)
65 +	3.34 (1.86)	3.59 (2.04)	2.04 (1.68)	2.45 (1.76)
Religion	***	***	***	***
Christianity				
Catholicism	4.49 (1.90)	4.56 (2.04)	3.26 (2.31)	3.61 (2.17)
Church of Jesus Christ of Latter-day Saints	4.86 (2.17)	4.97 (2.22)	4.11 (2.67)	4.19 (2.35)
Protestantism	3.64 (1.89)	3.81 (2.04)	2.33 (1.86)	2.78 (1.83)
Other Christian	4.24 (1.88)	4.18 (2.09)	2.93 (2.13)	3.29 (2.11)
Judaism	3.73 (1.93)	4.11 (2.34)	2.66 (2.08)	3.05 (2.00)
Islam	5.39 (1.90)	5.53 (1.98)	5.50 (1.99)	5.67 (1.74)
Buddhism	4.61 (2.00)	4.20 (2.09)	3.94 (2.69)	4.33 (2.42)
No Affiliation	3.82 (1.82)	3.96 (2.05)	2.55 (1.95)	2.93 (1.98)
Political Affiliation	*	**	*	**
Democrat	4.25 (1.98)	4.44 (2.12	3.13 (2.33)	3.48 (2.22)
Republican	4.07 (1.99)	4.01 (2.14)	2.77 (2.12)	3.17 (2.11)
Independent	3.86 (1.82)	4.06 (2.01)	2.69 (2.02)	3.06 (1.96)
No Affiliation	4.32 (1.56)	4.48 (1.86)	2.96 (1.99)	3.32 (1.90)
Race/Ethnicity	***	***	***	***
Caucasian or White	3.94 (1.90)^†^	4.01 (2.12)^†^	2.62 (2.09)^†^	2.96 (2.04)^†^
African American or Black	4.72 (1.72)	4.92 (1.80)	3.90 (2.15)^#^	4.33 (1.95)^#^
Latina or Hispanic	4.51 (1.81)	4.82 (1.89)	3.34 (2.22)	3.73 (2.05)
Asian	4.46 (1.75)	4.77 (1.77)	3.41 (2.06)	4.10 (2.02)
Indigenous or Native American	4.12 (1.69)	4.35 (2.12)	3.41 (2.18)	4.18 (2.16)
Native Hawaiian or Pacific Islander	5.29 (1.50)	3.43 (2.23)	3.00 (2.24)	4.00 (2.00)
Middle Eastern or North African Descent	4.57 (1.90)	4.57 (2.30)	3.00 (2.77)	3.57 (2.44)
Other	4.89 (2.21)	5.44 (1.59)	3.22 (2.59)	4.33 (2.35)
Income Level ($)	***	***	***	**
< 24,999	4.54 (1.86)	4.67 (1.99)	3.32 (2.20)	3.64 (2.12)
25,000–49,999	4.04 (1.77)	4.27 (2.02)	2.82 (2.07)	3.23 (2.03)
50,000–99,999	3.87 (1.93)	3.87 (2.07)	2.59 (2.03)	3.06 (2.04)
> 100,000	4.10 (2.05)	4.22 (2.22)	2.99 (2.38)	3.26 (2.23)
Education	**	N.S	N.S	**
Less than college	4.31 (1.86)	4.31 (2.04)	3.01 (2.15)	3.44 (2.08)
Trade school or associates’ degree	4.01 (1.89)	4.18 (2.04)	2.77 (2.11)	3.08 (2.05)
Bachelor’s degree	3.85 (1.93)	3.99 (2.10)	2.66 (2.08)	2.99 (2.08)
Advanced degree	4.08 (2.06)	4.26 (2.26)	3.05 (2.36)	3.51 (2.12)

1 – Strongly disagree, 2 – Disagree, 3 – Somewhat disagree, 4 – Neither agree nor disagree, 5 – Somewhat agree, 6 – Agree, 7 – Strongly agree

Group statistics calculated using one-way ANOVA test, *p < .05, **p < .01, ***p < .001

^†^Compared to all race/ethnicity groups, *p* < .001

^#^Compared to Non-White groups, *p* < .05

N.S., not significant

There were differences between the Christian religions for the requirement to continue treatment for the patient in the vignette (F(3, 858) = 11.08, *p* < 0.001), to perform CPR on the patient in the vignette (F(3, 858) = 7.49, *p* < 0.001), to request treatment for oneself (F(3, 858) = 12.41, *p* < 0.001), and to request treatment for a family member (F(3, 858) = 9.67, *p* < 0.001). Post hoc analysis showed that Protestant respondents had significantly less agreement on all four measures compared with all other Christian groups.

#### Political Affiliation

Furthermore, univariate ANOVA showed differences between political affiliations for the requirement to continue treatment for the patient in the vignette (F(3, 1383) = 2.87, *p* = 0.022), to perform CPR on the patient in the vignette (F(3, 1383) = 3.67, *p* < 0.01), to request treatment for oneself (F(3, 1383) = 3.10, *p* = 0.015), and to request treatment for a family member (F(3, 1383) = 2.47, *p* < 0.01). Post hoc analysis indicated that Democrats had stronger agreement to further treatment for themselves (${\overline{x} }_{diff}$ = 0.44, 95% CI 0.03–0.86), a family member (${\overline{x} }_{diff}$ = 0.43, 95% CI 0.02–0.83), and the patient in the vignette (${\overline{x} }_{diff}$ = 0.39, 95% CI 0.02–0.76) compared to Independents. Democrats also had greater agreement for further CPR for the patient in the vignette (${\overline{x} }_{diff}$ = 0.43, 95% CI 0.05–0.80) compared with Republicans (Table 4). However, even though Democrats had relatively more agreement for further treatment, their averages for further treatment for oneself and family member were between “somewhat disagree” and “neither agree nor disagree.” Similarly, Democrats’ averages for further treatment and CPR for the stranger were between “neither agree nor disagree” and “somewhat agree.”

#### Race and Ethnicity

There were differences between race and ethnicity for the requirement to continue treatment for the patient in the vignette (F(6, 1382) = 5.99, *p* < 0.001), to perform CPR on the patient in the vignette (F(6, 1382) = 7.64, *p* < 0.001), to request treatment for oneself (F(6, 1382) = 10.53, *p* < 0.001), and to request treatment for a family member (F(6, 1382) = 14.43, *p* < 0.001). Post hoc analysis showed that White participants had the least agreement to further treatment or CPR compared with all other races and ethnicities (Table 3). Among Non-White groups, those identifying as African American or Black had the most agreement for continued treatment for themselves and a family member, although their average responses were between “neither agree nor disagree” and “somewhat agree” (Table 4).

#### Income Level

There were differences in attitude across levels of income for the requirement to continue treatment for the patient in the vignette (F(3, 1383) = 7.21, *p* < 0.001), to perform CPR on the patient in the vignette (F(3, 1383) = 8.80, *p* < 0.001), to request treatment for oneself (F(3, 1383) = 6.96, *p* < 0.001), and to request treatment for a family member (F(3, 1383) = 4.41, *p* < 0.01). Post hoc analysis showed that the respondents in the lowest income bracket had more favorable attitudes toward requiring further treatment compared with those in the $25,000–$49,999 ($${\overline{x} }_{diff}$$ = 0.51, 95% CI 0.09–0.92), $50,000–$99,999 ($${\overline{x} }_{diff}$$ = 0.67, 95% CI 0.28–1.03), and > $100,000 ($${\overline{x} }_{diff}$$ = 0.44, 95% CI 0.03–0.85) brackets. However, the average score for respondents in the lowest income bracket was between “neither agree nor disagree” and “somewhat agree.” These differences remained significant after including quiz scores as a covariate.

#### Education Level

There were also differences between education levels for the requirement to continue treatment for the patient in the vignette (F(3, 1383) = 5.76, *p* < 0.001) and to request treatment for a family member (F(3, 1383) = 4.94, *p* = 0.02). Post hoc analysis showed that respondents with a Bachelor’s degree had significantly less agreement to further treatment for the patient in the vignette (${\overline{x} }_{diff}$ = − 0.46, 95% CI − 0.82 to − 0.10) and for treatment for a family member (${\overline{x} }_{diff}$ = − 0.53, 95% CI − 1.01 to − 0.04) compared with those with less than a college degree. There were no differences between levels of education in attitude for performing CPR on the patient in the vignette (F(3, 1385) = 1.22, *p* = 0.23) or requesting treatment for oneself (F(3, 1385) = 1.90, *p* = 0.066).

#### Linear Regression Model for Predicting Attitudes Toward Continued Treatment

Linear regression analysis was performed to investigate what variables predict response to requiring the hospital to continue treatment in the setting of brain death objection (Table 5). A backward variable selection yielded six independent variables: requesting further treatment for oneself, requesting further treatment for a family member, highest level of education, five-item knowledge score, male sex, and misunderstanding that the patient depicted in the vignette was alive according to U.S. laws (Table 5). The model predicted Likert scale responses for requiring continued treatment in the setting of brain death objection, F(6, 1380) = 142.74, adjusted *R*^2^ = 0.38, *p* < 0.001. The largest positive regression coefficients for predicting attitudes toward requiring further treatment were attitudes toward continuing treatment for oneself and continuing treatment for a family member after the determination of brain death. The largest negative regression coefficients were male sex and understanding that brain death is legal death. The model did not demonstrate collinearity with each factor’s tolerance > 0.10. The independence of residuals was demonstrated with a Durbin-Watson statistic of 2.03.

**Table 5:** Results of a multiple linear regression model for attitudes towards requiring further treatment for a brain-dead patient after family objection

Variable	B (95% CI)	S.E	β	*R*^2^	Δ*R*^2^
Model				0.38	0.38
Constant	3.84 (3.29, 4.40)***	0.28			
Agrees to further treatment for self	0.19 (0.12, 0.25)***	0.03	0.21		
Agrees to further treatment for family member	0.31 (0.25, 0.38)***	0.03	0.34		
Quiz score	− 0.16 (− 0.26, − 0.05)**	0.05	− 0.07		
Age > 65	− 0.26 (− 0.46, − 0.05)*	0.10	− 0.06		
Male gender	− 0.31 (− 0.46, − 0.02)***	0.08	− 0.08		
Understanding that brain-death is legal death	− 0.49 (− 0.70, − 0.27)***	0.10	− 0.10		

*B* = unstandardized regression coefficient; CI = confidence interval; S.E. = standard error of the coefficient; β = standardized coefficient; *R*^2^ = coefficient of determination; Δ*R*^2^ = adjusted *R*^2^

^*^p < .05, **p < .001, ***p < .001

## Deciding what Treatments Should be Offered and who Should Pay

Participants who agreed that the hospital should be required to continue treatment for a patient with brain death if the family requests it varied in what specific treatments they believed should be offered. Mechanical ventilation was the most common intervention selected, whereas noninvasive (e.g., wart removal) and invasive (e.g., intraabdominal surgery) were the least popular (Table 4). Of all the participants who correctly identified the patient as dead, more than half identified at least *some* form of intervention that should be offered, the most common being a mechanical ventilator.

Most respondents (71.6%) believed that the patient’s family should be required to pay for further treatment, with nearly 40% believing that private insurance should cover the cost (Table 4). However, there were differential answers depending on the political affiliation and income bracket. There were differences between income groups for requiring out-of-pocket (*χ*^2^ = 13.9; df = 3, *p* < 0.01) and government insurance (*χ*^2^ = 29.0; df = 3, *p* < 0.001). Differences between political affiliations were also present for requiring government insurance payment (*χ*^2^ = 16.7; df = 4, *p* < 0.01), and those with no political affiliation were more likely to require government insurance to pay for the continued cost of medical care (Supplemental Table 1).

## Discussion

Much of the literature on public attitudes toward brain death is based on the assumption that the public understands the basic facts of brain death.Although previous surveys help inform what clinicians ought to expect when talking to families with varying levels of understanding about brain death (e.g., an incorrect belief that brain death is reversible), it is not informative for a person’s genuine attitude (i.e., the thoughts and opinions one has after knowing the basic facts). Therefore, we provided a 2-min video that covers five basic facts of brain death and measured understanding with a validated tool. The average scores approached 90%, with most participants scoring 80% or better, which is comparable to medical trainees understanding similar basic facts of brain death [30–32]. Question 2 had the lowest number of correct responses, consistent with layperson responses during the development of the tool [26]. A recent study of the Canadian public showed that 67.2% of people believed that a patient with brain death depicted in a vignette was dead. In our study, which, in contrast to the Canadian study, included an educational component, 81.2% of participants correctly identified a similar patient as dead [28].

Furthermore, our data show that those with a higher education level (i.e., advanced degree) had lower scores on the five-item knowledge assessment than those with a lower education level. The technical aspects of brain death are not public knowledge. We should not assume that those with a higher education will inherently have a better understanding of brain death. Decision tools and informational pamphlets effectively inform families in the intensive care unit [33–35]. Our data support the continued development of educational tools that explain brain death in terms that are easily understandable to the general public at all levels of education.

Previous surveys have shown that most individuals would agree to the removal of medical support or organ procurement after the determination of brain death [9, 21, 27, 28]. However, none of these studies have measured attitudes toward continued care in the setting of a family member who does not accept the standard definition of death (i.e., that brain death is death). Nearly half of all respondents in our survey at least agreed that the hospital should be required to continue treatment if the family rejects brain death and requests further intervention, which is similar to health care professionals who are exposed to patients with severe brain injury [36]. This attitude is not entirely explained by a lack of understanding of brain death, as nearly a quarter of all respondents who correctly identified the patient as dead would agree to further treatment if the family requested it and CPR if the patient’s heart stopped beating. This amounts to a quarter of people believing that medical interventions should be offered to what they understand to be a legally dead body. However, understanding a medico-legal standard does not entail an agreement with that standard. Therefore, it is reasonable to assume that some of these respondents who would continue medical support after brain death despite an understanding of its medico-legal standing do so because they believe that brain death is not equivalent to death. Nearly a quarter of participants would request continued treatment for themselves after a determination of brain death. This is higher than the previously reported 18.6% of respondents who believed their own death should be determined solely by cardiopulmonary standards [37].

Furthermore, when presented with a patient with brain death who had been receiving continued support for 8 months due to family objection to brain death, 16% of U.S. neurologists responded “no” when asked if the patient had been dead during this period [38]. These data represent the inherent tension of the death debate: who ought to decide what is or is not the correct view of death? There are many arguments for and against recognizing brain death [39–42]. Many of these arguments rest on differences in principles (e.g., the essential functions of life) that rely on metaphysical assumptions, not clinical data. For example, Veatch [40] and Ross [43] argue that when there are reasonable disagreements, such as in the death debate, we ought to endorse pluralism in our laws, thereby allowing individuals to choose between brain death, cardiopulmonary death, and higher-brain death (i.e., irreversible loss of consciousness). Although our study did not investigate attitudes toward higher-brain death, it does suggest that there is substantial public support for a limited pluralistic UDDA in which a patient ought to receive continued support if they reject brain death, and a substantial percentage of the public would want to invoke a conscience clause for themselves or a family member.

Our data align with previous data on the differential attitudes toward end-of-life care between White and Non-White patients. In one retrospective study, surrogates of Non-White patients were 44% less likely to withdraw mechanical ventilation compared to surrogates of White patients [44]. Our study did not investigate the *reasons* why Black and African American participants were more likely to agree to further treatment for a stranger, themselves, and a family member. Commentators have used the case of Jahi McMath to demonstrate the intersection of race and medical decision-making and how mistrust in the medical system influences decisions at the end of life [45, 46]. However, although barriers in trust, breakdown in communication, and health literacy have been proposed as reasons for the differential attitudes among minority populations for continuing medical support for patients with traumatic brain injury, it is unclear what reasons inform different attitudes toward a legal exemption for opting out of brain death. Minority patients already have to navigate a medical system in which they have been marginalized, and no decision has comparable significance as the determination of death. Professionals in the intensive care setting ought to practice cultural sensitivity and be aware of how past medical trauma and mistrust within minority populations influence attitudes toward opting out of brain death determination. Morrison and Kirschen distinguish three reasons for objecting to brain death: informational, emotional, and principled [11]. Future investigation into the prevalence of the reasons why families opt-out of brain death will shed light on whether educational efforts (i.e. to address informational reasons) or time (i.e. to address emotional reasons) will affect whether an individual ultimately chooses to opt-out. Our data show that the differences between religions across all four attitude measures were maintained even after using the five-item quiz score as a covariate. This suggests that truly principled reasons (e.g., religious objections) are unlikely to be affected by additional education or time. Understanding these perspectives is crucial for any discussions about potential legislative changes to the UDDA. Future research should continue to explore the underpinnings of these perspectives, particularly among minority groups who have historically expressed distrust toward the medical system.

There was a variety of interventions that participants believed should be offered to a patient with brain death should their family request continued treatment. Although the most common answer was a mechanical ventilator, nearly one third stated it should not be offered. Mechanical ventilation is required for continuous oxygenation for individuals with brain death, and therefore required for continued cardiopulmonary function. To agree that further treatment should be provided but disagree that mechanical ventilation should be offered is inconsistent with honoring a patient’s preference for continued treatment. Although many of our respondents had a logical inconsistency in their response, we think that even a small correction by a clinician to remind them of the importance of the ventilator for continued function would make most of them change their response and agree to requiring a ventilator. Approximately one third of the sample would offer nutrition and hydration after a determination of brain death. This contrasts with U.S. neurologists, in which 4% in the nonaccommodation states and 7% in the four accommodating states (i.e., New Jersey, New York, Illinois, and California) would start nutrition for a patient with brain death whose family had a religious-based objection to brain death. A similar discrepancy between neurologists and our participants is seen with hydration, in which only 10% in nonaccommodating states and 16% in accommodating states would start intravenous fluids after a determination of brain death [47]. Tensions may arise between families and medical staff on what is appropriate medical intervention, especially in the context of brain death, in which for some families the decision to withhold certain treatment (e.g., nutrition) could be a matter of life and death. Our data suggest that what neurologists deem as a medically inappropriate treatment that should not be offered after a determination of brain death is deemed medically appropriate by many laypersons. Such disagreements may result in fractured relationships between providers and families in the intensive care unit, and medical professionals ought to be prepared to explain the clinical indications for various interventions in the setting of brain death.

The decision to continue medical interventions after a determination of brain death incurs considerable medical costs. When an organ donor is declared brain dead, there may be a short period of time in which medical interventions are continued in order for the organ procurement organization and hospital to arrange recipients and prepare for procurement. The patient’s family is not responsible for costs accrued after the determination of brain death. Instead, these expenses are often distributed between the organ procurement organization, transplant center, and federal government. However, in the case of brain death objection, the continued treatment does not serve a public good (i.e., organ procurement) but rather serves to respect the religious or philosophical views of the patient and their family. This may explain why, in our survey, the majority of participants answered that the patient’s family should pay for further treatment. Insurance companies are required to cover the continued cost of care for families in New Jersey who opt-out of brain death [48]. It is currently unclear what treatments will be offered, who will cover the costs of treatments, and how these resources will be allocated between patients in a world where the UDDA includes a conscience clause. Our data suggest that there would be widespread disagreement if policymakers required private or government insurance to cover such continued costs.

Our study has several limitations. The translation of survey responses to real-world decisions is limited because an individual may believe that they would not continue further treatment for a family member, but when they find themselves in the intensive care unit being called on to make a decision, their attitude may be different. Although 99.7% of participants indicated that they had watched the video, we were not able to generate analytics to independently verify affirmative confirmations, which limits our ability to assess the video’s impact on their decision-making fully. In addition, similar to any incentivized survey, there is a potential limitation in which some respondents may have participated mainly for the reward. In this study, the rewards ranged from $1 to $2.57, which could have influenced the level of engagement or attention given to the survey. This will foster a more inclusive and informed debate about end-of-life care. Despite including an educational video, only 68% of participants who would require further treatment chose an option (e.g., mechanical ventilation) that would allow the patient’s heartbeat to continue. Although educational materials such as the video we used may be effective at educating a wide audience on the basic facts of brain death, they do not replace lengthier, in-depth discussions between family members and attentive intensive care staff. Our sample was skewed toward individuals whose highest level of education is a high school degree, and although the sample of White and Black or African American approximated national averages, Latino or Hispanic ethnicity was underrepresented in our study. Although there was diversity across multiple demographic variables, the study population was limited to those who were enrolled through Centiment. Therefore, it is possible that our results do not reflect the population as a whole. Lastly, grouping participants by major religion is only an approximation of their beliefs, as there can be a variety of views on brain death within one religion [49–51]. For example, interpretations of the Talmud have led to differing views within Judaism [52, 53], and there is no consensus view within Islam [54]. These groups were a small proportion of this study. Further investigation into these particular groups may help policymakers better understand the most common religious reasons for denying brain death as death.

## Conclusions

After nearly 40 years of being the model law for death determination, the ULC revisited the UDDA to investigate whether a legal exemption for personal belief in brain death determination would be an appropriate addition. Our results show that there is a substantial proportion of the population that would agree to further treatment for themselves and a family member after a declaration of brain death and that an even larger proportion would require the hospital to continue treatment for a stranger whose family objects to brain death. The present study provides insight into the public’s perception of opting out of brain death. It is informative for physicians, lawyers, bioethicists, and policymakers at the national level when deliberating over revisions to the UDDA.

## Supplementary Information

Below is the link to the electronic supplementary material.

Supplementary file1 (DOCX 18 KB)

Supplemental Figure 1. (A) Distribution of agreement for whether a hospital should be required to continue treatment after a determination of brain death if the family member requests it, (B) whether CPR should be performed after a determination of brain death if the family member requests it, (C) agreement for wanting further treatment for oneself after a determination of brain death, (D) agreement for requesting further treatment for a family member after a determination of brain death.

Supplementary file3 (DOCX 14 KB)
