# Supplementary material for: An Investigation into the Public’s Attitude Toward Opting out of Brain Death
Source: Neurocrit Care. 2025 Jan 14;43(1):262–76. doi: 10.1007/s12028-024-02196-8 (PMC12321666; doi:10.1007/s12028-024-02196-8)
Supplement: Supplementary file 1 — Supplementary file1 (DOCX 18 KB) [file 12028_2024_2196_MOESM1_ESM.docx]

**Please read the following for important information regarding this study.**

**Introduction**
You are being asked to participate in a research study conducted by Nicholas Ludka, Medical Student at Oakland University William Beaumont School of Medicine, Dr. Abram Brummett, Assistant Professor of Foundational Medical Studies at Oakland University William Beaumont School of Medicine, and Dr. Deidre Hurse, Assistant Professor of Foundational Medical Studies at Oakland University William Beaumont School of Medicine.
 
Your decision to participate in this study is voluntary. You can choose to stop your participation at any time or skip any part of the study. Your decision will not affect your present or future relationship with Oakland University, the researchers, or Oakland University William Beaumont School of Medicine. 
 
**What is the purpose of this study?**
The purpose of this research study is to assess the public’s knowledge of brain death and attitudes toward the ability to opt out of brain death.
 
**Who can participate in this study?**
You are being asked to participate in the study because you are a member of the United States adult population.
 
**Who is the financial sponsor for this study?**
This study is funded by the Department of Foundational Medical Studies at Oakland University William Beaumont School of Medicine and the Black Faculty Association at Oakland University. The research team members have no conflicts related to this funding**.**
 
**What do I have to do?**
You will be asked to watch a 2-minute video focused on the basic medical and legal facts that surround brain death. You will then take an anonymous online survey about your understanding of brain death as well as your attitudes toward the ability to opt out of brain death.
 
**Are there any risks to me?**
For this study, the potential risks are potential emotional disturbance from watching an animated video about brain death and reading about a case of an individual who is brain dead.
 
**Are there any benefits to me?**
*Although there may be no direct benefits to you, the results of this study may benefit others in the future.*
 
**Will I receive anything for participating?**
In consideration of your time, the outlined reward will be provided upon successful completion of the survey.
 
**What if I want to stop participating in this study?**
*If you want to stop participating, close your browser before clicking ‘submit.’ If you click ‘submit,’ it will not be possible to stop participating.*
 
**Who can I contact if I have questions about this study?**
Dr. Abram Brummett, abrummett@oakland.edu 
Dr. Deidre Hurse, dhurse@oakland.edu  
Nicholas Ludka, nludka@oakland.edu
 
**For questions regarding your rights as a participant in human subject research, you may contact the Oakland University Institutional Review Board, 248-370-4898.**

**Validated Tool for Testing Understanding of Brain Death**

Question: Can someone who is brain dead breathe without the support of a breathing machine?

-Yes

-No

Question: Can someone who is brain dead ever wake up (recover)?

-Yes

-No

Question: Will someone who is brain dead react (grimace, move away, or blink) if someone touches their eyeball?

-Yes

-No

Question: Can a person be brain dead even if their heart is still beating?

-Yes

-No

Question: Is brain death different from a coma or vegetative state?

-Yes

-No

**Vignette and Attitude Items**

C.H. is a patient in the intensive care unit at the local hospital. Three days ago, C.H. suffered a significant brain injury. A breathing machine is keeping C.H.’s lungs working, and medications are being used to maintain C.H.’s other organs. A neurologist is consulted to determine the extent of C.H.’s brain injury. The neurologist’s examination shows that C.H. is unconscious, cannot breathe without the ventilator, and doesn’t have any brainstem reflexes. Based on these results, the neurologist determines that C.H. is brain dead, and, further, that C.H. will never recover any of these functions. When the neurologist meets with C.H.’s family to tell them that C.H. is brain dead, the family objects and says that C.H. isn’t dead until the heart stops beating.

Question: Is C.H. dead according to current U.S. laws?

-Yes

-No

Question: The hospital should be required to continue treatment if C.H.’s family doesn’t believe that brain death is the same as death.

-Strongly agree

-Agree

-Somewhat agree

-Neither agree nor disagree

-Somewhat disagree

-Disagree

-Strongly disagree

Question: Who should have to pay for the continued care of C.H.? (Select all that apply)

-C.H.’s family (out-of-pocket payment)

-Private insurance

-Government insurance (Medicare or Medicaid)

-The hospital

Question: If C.H.’s heart stops beating, the medical team should perform CPR if the family requests it.

-Strongly agree

-Agree

-Somewhat agree

-Neither agree nor disagree

-Somewhat disagree

-Disagree

-Strongly disagree

Question: Which of the following treatments should be offered to C.H. after the determination of brain death? (select all that apply)

-Mechanical ventilator (breathing machine)

-Nutrition

-Hydration

-Medications

-Non-invasive procedures (e.g., teeth cleaning, wart removal)

-Invasive procedures (e.g., gallbladder removal, organ transplant)

Question: If I was declared brain dead. I would want treatment to be continued so long as my heart kept beating.

-Strongly agree

-Agree

-Somewhat agree

-Neither agree nor disagree

-Somewhat disagree

-Disagree

-Strongly disagree

Question: I would object to brain death determination for a family member and request that treatment be continued so long as their heart kept beating.

-Strongly agree

-Agree

-Somewhat agree

-Neither agree nor disagree

-Somewhat disagree

-Disagree

-Strongly disagree

**Demographic Information**

Question: What is your 5-digit zip code?

Question: What is your gender identity?

-Male

-Female

-Transgender

-Other

Question: During the past year, what was the total combined income for your household?

-Less than $24,999

-$25,000-$49,000

-$50,000-$99,999

-Greater than $100,000

Question: What is the highest degree or level of education you have completed?

-Less than high school

-High school diploma or equivalent

-Trade school

-Associates degree (e.g. A.A., A.S.)

-Bachelor’s degree (e.g. B.A., B.S)

-Master’s degree (e.g., M.A., M.S., M.Ed.)

-Doctorate degree (e.g., Ph.D., Ed.D.)

-Professional degree (e.g., M.D., D.O., D.V.M.)

Question: What is your age in years?

-(write in)

Question: What is your political affiliation?

-Democrat

-Republican

-Independent

-Other (write in)

-No affiliation

Question: With what religious institution are you affiliated?

-Protestantism

-Catholicism

-Other Christian

-Church of Jesus Christ of Ladder-day Saints

-Judaism

-Islam

-Buddhism

-Other (write in)

-No affiliation

Question: How committed are you to the teachings of your religion?

-Not committed

-Slightly committed

-Moderately committed

-Very committed

Question: Please specify your ethnicity (Check all that apply)

-African American or Black

-Asian

-Caucasian or White

-Indigenous or Native American

-Latin or Hispanic

-Native Hawaiian or Pacific Islander

-Middle Eastern or Northern African Descent

-Other (write in)
