# Supplementary figures and images for: An Investigation into the Public’s Attitude Toward Opting out of Brain Death
Source: Neurocrit Care. 2025 Jan 14;43(1):262–76. doi: 10.1007/s12028-024-02196-8 (PMC12321666; doi:10.1007/s12028-024-02196-8)

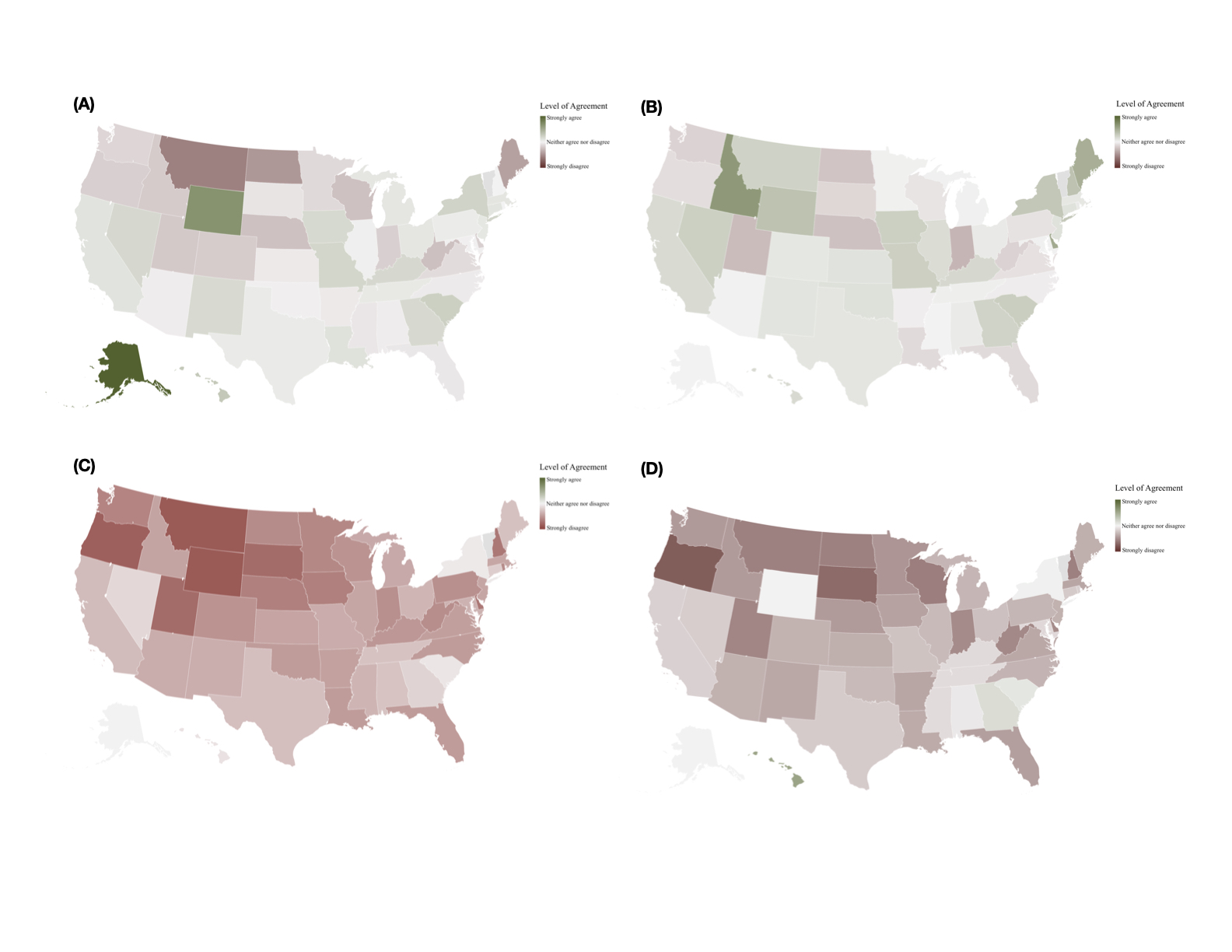

Supplement: Supplementary file 2 — Supplemental Figure 1. (A) Distribution of agreement for whether a hospital should be required to continue treatment after a determination of brain death if the family member requests it, (B) whether CPR should be performed after a determination of brain death if the family member requests it, (C) agreement for wanting further treatment for oneself after a determination of brain death, (D) agreement for requesting further treatment for a family member after a determination of brain death. [file 12028_2024_2196_MOESM2_ESM.jpeg]
