# Supplementary material for: An Investigation into the Public’s Attitude Toward Opting out of Brain Death
Source: Neurocrit Care. 2025 Jan 14;43(1):262–76. doi: 10.1007/s12028-024-02196-8 (PMC12321666; doi:10.1007/s12028-024-02196-8)
Supplement: Supplementary file 3 — Supplementary file3 (DOCX 14 KB) [file 12028_2024_2196_MOESM3_ESM.docx]

**Supplemental Table I.** Types of payers that should be required to cover the cost of continued treatment by political affiliation and income bracket.

|  | Out-of-pocket | Private insurance | Government insurance | Hospital |
| --- | --- | --- | --- | --- |
| Political affiliation, % |  |  |  |  |
| Democrat | 68.3 | 40.0 | 33.5* | 10.0 |
| Republican | 70.4 | 42.4 | 25.7 | 9.1 |
| Independent | 74.1 | 34.9 | 26.7 | 7.8 |
| No affiliation | 70.2 | 37.2 | 42.6 | 11.7 |
|  |  |  |  |  |
| Income bracket, % |  |  |  |  |
| <24,999 | 61.6* | 38.1 | 42.2* | 11.9 |
| 25,000-49,999 | 71.4 | 40.0 | 29.8 | 9.2 |
| 50,000-99,999 | 72.7 | 40.9 | 23.4 | 9.2 |
| >100,000 | 74.5 | 37.6 | 29.3 | 7.6 |
| Statistics calculated using Chi-square test, *p<.05, **p<.01, ***p<.001 | | | | |
